# Solid-waste management in Jalandhar city and its impact on community health

**DOI:** 10.4103/0019-5278.43265

**Published:** 2008-08

**Authors:** Avinash Puri, Manoj Kumar, Eonkar Johal

**Affiliations:** Postgraduate Institute of Medical Education and Research, Chandigarh - 160 012, India

**Keywords:** Jalandhar, pollution, solid waste, vector-borne diseases

## Abstract

In this study, solid-waste management practices were evaluated in order to find out its link with occurrence of vector-borne disease. Strategies for solid-waste management were employed as practical model to solve the problems regarding pollution which is originated by solid-waste.

## INTRODUCTION

Solid waste (SW) can be defined as the material that no longer has any value to the person who is responsible for it and is not intended to be discharged through a pipe. It does not normally include human excreta. It is generated by domestic, commercial, industrial, healthcare, agricultural and mineral extraction activities and accumulates in streets and public places.[1] The words ‘garbage,’ ‘trash,’ ‘refuses’ and ‘rubbish’ are used to refer to some forms of SW.

### Status of solid-waste management in Jalandhar

In this comprehensive investigation, Jalandhar city was chosen as a model region of the Punjab province. Besides a dwelled suburban area, the population of this region is around 20 00 000. The city is located almost 375 km from Delhi and about 90 km from Amritsar. Jalandhar is named after Jalandhar, a demon king who lived in water, as suggested by his name. If the name is divided in two parts - JAL and DHAR - JAL means water and DHAR means to live in. The spread of education cannot be overlooked while assessing the progress of a particular society.[2] It is correct to say that the spread of education, language and literature are responsible for the development of Jalandhar district. Only educated and conscious people can bring about any development. Jalandhar, with the passage of time, is being known as the media centre of Punjab. Here is a large industry that manufactures sports material and other products. The sports products are exported in a larger quantity. Jalandhar falls in the Doaba region, comprising the districts Jalandhar, Hoshiarpur and Kapurthala. The Doaba region is surrounded by the rivers Beas and Sutlej. It has an urban population of almost a million, and another million live in the rural areas outside the city.

### Sanitation set up in the Municipal Corporation of Jalandhar

The Health Officer heads the sanitation operation of city supervision of the city. Two chief sanitary inspectors and four sanitary workers, Safai Sewaks, work under the Supervisor.

The organization setup with the number of employees at each level is given below [Box 1].

**Table:** 

Portfolio	No. of officers
Health officer	1
Chief sanitary inspector	2
Sanitary inspector	4
Safai sewak	1400
Safai sewak truck collie	150
Drivers	32
Drivers (three wheelers)	35
Total	1624

### Generation of solidwaste

The sources of SW are residential, commercial institutional, constructional and demolition, municipal service, industrial, agriculture and dead animals.[3–5] The distribution of the area of the city of Jalandhar in various zones is as shown below:[6]

1. Residential zone - 85%,
2. Industrial zone - 10%,
3. Mixed zone - 5%,
4. No. of large and small factory units - 3500,
5. No. of trade units - 1050,
6. No. of shops - 35 000,
7. No. of tanneries - 30,
8. No. of electroplating industries - 2,
9. No. of private hospitals - 50 and
10. No. of health centres - 10.

A total of 300-400 tonnes of garbage is collected and disposed off daily in the city from the above-mentioned sources.[7]

**Table:** Physical characteristics based on wet basis (%)[8]

Parameters	Average	Range
Metal (ferrous)	0.01	0.00-1.00
Metal (nonferrous)	0.13	0.00-0.04
Earthware stone, etc.	5.17	0.50-15.00
Glass/ceramics	0.57	0.00-2.10
Fine earth	24.15	19.92-25.72
Paper/cardboard	3.43	0.2-10.80
Wooden matter	0.09	0.00-0.30
Rags	3.95	0.10-9.80
Rubber, leather	1.31	0.00-4.00
Plastics	7.42	3.20-14.5
Moisture content	44.53	6.90-68.10

**Table:** Average chemical characteristics

Parameters	Average
pH	8.04
Calorific value	1616.18
Compostable matter	53.75
Nonvolatile matter (%)	70.05
Volatile matter (%)	29.95
Nitrogen (%)	2.51
Phosphorous (%)	0.31
Potassium (%)	0.49
Organic carbon (%)	10.38
Hydrogen (%)	1.20
C/N	4.13

The waste is 85% nonhazardous, 10% infectious and 5% noninfectious. It is 54% compostable, 64% combustible, 24% inert and 11% recyclable.[9]

### Status and analysis of functional elements

The functional elements of SW management in this city include Waste Generation, Collection, Transportation, Processing and Final Disposal.

**Waste generation:** For efficient SW management, study of the generation rate is very important. The generation rate varies from activity to activity and the exact generation rate must be determined after averaging over the time factor and including several of the waste producers.[10] The average waste generated in Jalandhar city is 350 TPD. In all, there are about 320 garbage bins placed in city. Garbage is finally disposed off in an open dump at Suchipind village on Hoshiarpur road and Wariana on Kapurthala road. Garbage-lifting vehicles involved in this operation are:[11]

**Table:** 

Type of vehicle	Number
Truck tipper	7
Tractor trailer	5
Refuse collector	2
Dumper placer	5
Tricycle	300
Wheel barrow	200

**Table:** Sources of waste generation [Box 2][12]

Name	Content	Sources
Garbage	From cooking food and domestic work contents	House holds and hotels, etc.
Rubbish	Markets refuse rags, cloth and leather	Stores and markets, etc.
Ashes	Residue form	Fire
Bulk waste	Large auto parts, gyres, etc.	Service station
Street refuses	Dust and dirt	Street sweepings and litter, etc.
Special waste	Hazardous waste	Hospitals and industry

### Onsite handling, processing and storage

In Jalandhar city, most of the habitable/residential areas have limited storage spaces. In these areas, the waste is of mostly of a biodegradable nature. In some places, open dumping of the garbage is noticed, which causes health hazards as well as fly nuisance.

**Handling:** It refers to the activities associated with managing SW until they are placed in the containers used for their storage before collection or return to drop off and recycling centres.

There are various problems that could be related to handling and storage of SW are seen, like there are a number of places where there are dumps of SW and thus these points if unattended create small nuisance and health hazards. Stray animals like pigs, dogs and cows further aggravate the problem of spreading and littering of SW as they are seen at the site of handling and storage of SW in the study area.

**Solidwaste segregation:** SW is not segregated; rag pickers collect SW from the streets, bins and deposit sites. Storage spaces are not often adequate. People drop the SW outside the bins.[13]

**Collection:** SW is collected from the bins from every point and collection from residential areas is carried out daily as the organic matter decomposes rapidly due to a hot climate.

The World Health Organization recommends that collection of SW should be carried out twice a week from bins in Jalandhar city.[8] Collection is only performed when the bins are filled up. Hand driver cart pullers collect the SW from door to door. These cart pullers segregate the plastic bags, polythene and metal, which is then sold to the kabariwalas. By this, nondegradable solids are separated from organic substances. This collection system is also economically feasible.

**Transportation:** Transportation means ‘transfer’ of SW from the storage place to the dumping ground. For this purpose, vehicles are dependent on the physical layout of the roads and the cost of manpower available, maintenance provisions, truck tippers, tractor trailer, etc. that are used for final transportation of SW to the site. About 350 TPD waste is generated on a daily basis.

### Recycling process and recovery recycling process

The Municipal corporation, Jalandhar, has signed a MoU with the Punjab Grow More Fertilizers Ltd. A plant has been set up at the village warrians Basti Bawa Khel. It was earlier the dumping site of MCJ. By this process, organized SW is converted into manure by the process of composting. This site has 14 acres of land having a capacity of 600 TPD. About 100 TPD SW is transferred to the warraina site. Nearly 250 TPD is dumped at the Suchi-Pind site.

In order to utilize the entire SW produced in the city of Jalandhar for making organic manure, the following two requirements must be met:

- More land is required at the dumping yard with a provision that it can used for at least 30 years and
- Proper marketing by government so that the organic manure produced should be sold out. As 300 TPD SW is produced per day, 150 TPD of organic manure is thus generated. Therefore, marketing 150 TPD organic manure should be planned.[14]

### Waste disposal options

A number of waste disposal options are available in the form of repetitive disposal technology. Table 3 below lists the leading ones along with the equipments required for them [Box 3].

**Table:** 

Technology	Major equipment
Sanitary landfills	Reactors/digesters, synthetic liners, pumps
Fuel pelletization	Pelletization equipment
Composting	Mechanicial composters

**Sanitary landfilling:** SWs are placed in the sanitary landfill system (trenches, pits) in alternate layers of 80 cm-thick refuse and then covered with an earth fill of 20 cm thickness. After 2-3 years, the SW volume shrinks by 25-30% and the land can be used for parks, roads or as land for small buildings with normal compaction. A landfill site can take 500 bags of refuse per cubic meter of trench space available. Care should be taken while locating the site for dumping refuse as the land should be selected after taking into account that it can be used for 25-30 years. Land filling depends on the availability of land area, soil conditions, grand water table, topography, distance from the residential area and ultimate usage of site after reclamation.

The landfill operation is a biological method of waste treatment. SW can be stabilized by dividing it into five distinct phases with the overall process. In the first phase, aerobic bacteria deplete the available oxygen as a result of aerobic respiration and the temperature increases. In the second phase, anaerobic conditions become established and hydrogen and carbon dioxide is evolved. In the third phase, methane is librated and in the fourth phase, methonogic activities become stabilized. In the fifth phase, the system returns to aerobic conditions within the land fill. The duration of each phase varies with the environmental conditions.

**Biomethane technology:** This process is used for the production of methane from the SW. In this, first of all separation and size reduction of the SW is carried out. After this, moisture and nutrients are added. The pH is adjusted to about 6.7 and temperature of the slurry is increased to 55-60°C. The slurry is mixed well for about 7-10 days. After this, storage of the gas is carried out.

**Incineration:** Incineration involves the burning of the SW at very high temperatures. In this method, the volume of the SW is reduced up to 90%. The unburnt SW, which is left, is about 25% of the original waste.

**Composting:** This method is an aerobic method of decomposition of the SW. Many types of microorganisms already present in the waste stabilize the SW. The organisms include bacteria, which predominates at all stages, fungi, which appear after the first week and actionomycetes, which assist during the final stages. Mesophillic bacteria present oxidize the organized matter in the refuse to CO_2_ and release it as heat. The temperature increases up to 45°C. Thermophillic bacteria take over and continue the decomposition. The temperature further increases to 60°C. After this, the SW is turned. After about 3 weeks, the composites are stabilized.

### The Municipal Corporation, Jalandhar, has signed a MoU with the Punjab Grow More Fertilizers Ltd. for converting waste into manure using the waste sanitization treatment

**Waste sanitization treatment method:**[15] SW is first of all treated with biological inoculum at the collection point. The celrich substrate DF-BC-01 (manufacturer USA) is a mixture of biological enzymes and herbal extracts that is spread over the SW. The material is nonhazardous and nontoxic. The SW becomes free of hazardous pathogens, which eliminates the foul smell from the SW. Dumping points get hygienically upgraded. The SW becomes free of flies, insects and other disease-carrying vectors. This provides better working conditions and reduces the chances of smoke, fire and explosion hazards at the dump yards as the production of methane is reduced by this.

### Advantage of the technology

- Area required in this process is very less.
- Corporation gets an annual lease rent as well as royalty to meet the collection cost partially.
- Clean refuse is generated, which can be used for landfilling.
- Polythene and plastic material that is segregated can be recycled.

### **Use of plastics and their recycling**[16]

Plastics, due to its advantages like its durability, lightness, and ease to be moulded, is used everywhere, for example:

**In domestic purposes:** As carry bags, pet bottles, trash bags, containers.

**In air, road, rail travel:** As cold drink or mineral water bottles, plastic plates, cups.

**In hospitals:** As glucose or other IV fluid bottles, disposable syringes and injections, catheters, wine bags, gloves.

**In shops and hotels:** As packing items, plastic bags and disposable utensils.

Plastic contains a certain component called dioxin that is highly toxic and carcinogenic. After burning, especially PVC, it releases this dioxin and also furan into the environment. To avoid health hazards, plastics should be recycled.

**Graph 1:**
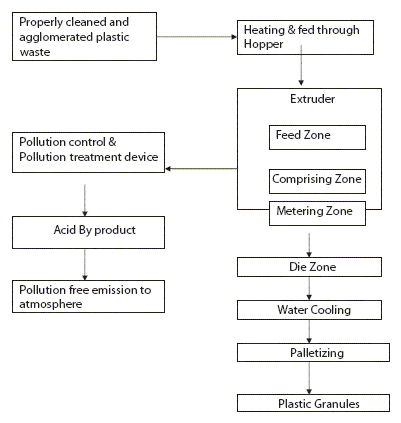
Green recycling process

Plastic recycling can be performed by the ‘Green Recycling Process.’ A sketch of the pilot used for this purpose is given below [Graph 1].

### Health impact of solidwaste[17]

As SW is a major part of environment pollution, it is responsible for spreading many harmful and infectious diseases. The increase in SW is due to an increase in the population. As the population increases, the demand for food and other essentials also increases such that waste is also increased. Some people throw this waste into streets, roads and at other public places, which attracts flies, insects, rats etc., which helps in spreading the diseases. Unattended waste is normally wet and has a bad odour due to decomposition. This type of waste leads to epidemics in various parts of the country.

- Domestic waste decomposes and spreads diseases.[18]
- Waste from agriculture and industries can also cause serious health diseases because these wastes may include some chemicals, pesticides, metals etc.
- Uncollected SW may also affect water bodies. When water gets infected, it causes many water-borne diseases the to surrounding community. From this SW everyone gets affected. e.g. collectors of this waste material and persons working in various shops or factories.
- Like in an industry where some chemicals are used, certain chemicals, if released untreated, e.g. cyanides, mercury and polychlorinated biphenyls, are highly toxic and their exposure can cause severe disease or also death.
- Plastic is also very harmful to the health. The unhygienic use and disposal of plastic causes very toxic effects as some coloured plastics contain heavymetals that are very toxic, e.g. copper, lead mercury, lead, chromium, cobalt, selenium and cadmium.
- As we know that there is manual collection of SW from door to door, persons collecting this SW are exposed to many diseases and infections.

**Health impact assessment:** A number of risk factors in the form of foetal diseases are associated with the malpractices of SW management. The potential diseases that were identified in the area of MCJ may occur due to the prevailing SWM methods.[19]

The SW-related vector-borne diseases identified are malaria, dengue, Kala Azar, fever and loose motion.

The worst affected areas that are closer to the dumping sites were also selected. These are the two villages, namely Suchi village and Waryana village. All these locations are very close to the dumping sites of MCJ. One more area that is away from the dumping sites, ‘Pucca Bagh’ was also selected.

A questionnaire was prepared keeping in mind all the environmental indices and the impact assessment methodology and established strategies. It was circulated among the residents of these villages originally in the local language ‘Punjabi’ to collect feedback from them. The modes of survey carried out for getting feedback from these villages is as under:

- Warriana: A random survey was conducted for 150 persons in the Wariana village. This village is about 5 km from the wariana dumping site and composting plant. In this village, 3.5% had malaria 0% from dengue, 0% from Kala Azar, 90% from fever and 95% from loose motions.
- Suchi Pind village: A random survey was conducted for 150 persons in the Suchipind village. This village is about 1 km from the Suchipind dumping site. In this village, 1.2% had suffered from malaria, 0% from dengue, 0% from Kala Azar, 92% from fever and 94% from loose motions.
- Pucca Bagh: A random survey was conducted for 150 persons in the Pucca Bagh area. This area is located in centre of Jalandhar city where proper SW management is performed. SW is collected properly from each house. In Pucca Bagh,.9% people suffered from malaria, 0% from dengue, 0% from Kala Azar, 50% from fever and 52% from loose motions.

The received feedback data were compiled and analyzed using the established statistical methods. The findings related to the vector-borne diseases caused by SW are summarized in Tables 1 and 2. Table 2 provides the percentage extent of problems caused by the agents and vectors due to malpractices of SW management adopted in these areas.

**Table 1:** Results of the survey conducted for solidwaste-related vector-borne diseases

Vector-borne diseases	Wariana (*n* = 150)	Suchi pind (*n* = 150)	Pucca bagh (*n* = 150)
Malaria	5 (3.5)	2 (1.2)	1 (0.9)
Dengue	0 (0.0)	0 (0.0)	0 (0.0)
Kala Azar	0 (0.0)	0 (0.0)	0 (0.0)
Fever	135 (90)	138 (92)	75 (50)
Loose motion	142 (95)	141 (94)	78 (52)

Figures in paranthesis are in percentage

**Table 2:** Household perception of the impact of malpractices of SWM

Household perception	Waraina (*n* = 150)	Suchi pind (*n* = 150)	Pucca bagh (*n* = 150)
Solidwaste pollution	16.0	8.0	10.0
No problem	-	-	-
Problem	92.0	90.0	52.0
Major problem	2.0	3.0	2.0

Figures are in percentage

In order to ascertain that the higher degree of infections in the villages of Suchi Pind and Waraina are only because of the impact of SW, a survey was also conducted for the smoking and drinking habits of the residents of the three locations, i.e. Suchi Pind, Waraina and Pucca Bagh.

It is clear from the data that occurrence of dengue and Kala Azar is not observed in any of the locations; however, other diseases like malaria and loose motions have been reported. A large number of residents, up to 90-95%, were found suffering from fever and loose motions more than once every year. This is indicative of a strong to moderate health impact on the resident population due to the SW being dumped in their vicinity. There is no established correlation of occurrence of these infections due to the smoking and drinking habits of the residents of these areas. Thus, the sole cause of these illnesses lies in the faulty disposal of the wastes closer to these locations.[20]

### How can we prevent these diseases

- Proper disposal: A proper method should be used to dispose SW because unattended waste is responsible for spreading these infectious diseases.
- By avoiding water pollution: SW collection and disposal should be away from a water supply or a big water body because, as we disposed earlier, that there are many diseases that can spread by water pollution, e.g. cholera, typhoid, diarrhoea, etc. Hence, village waste should not be dropped near wells or taps etc. The wells should be covered.
- All the eatables should be covered so that flies cannot infect them. Boiled water should be used for drinking and other purpose in the pitches.
- Early diagnosis and treatment: There should be different surveillance programmes arranged in urban and rural areas, e.g. the malarial surveillance programme. Also, to detect the symptoms of a patient, various diagnostic tests should be carried out as soon as possible so that proper treatment can be started.
- Isolation of the patient having a contagious disease: Isolation of the patient is necessary to avoid further spread of the disease and to avoid major problems like epidemics. Vaccinations of some contract calls should be performed according to the severity of the disease.

## DISCUSSION

The problem related to SW management and its heath impact is investigated in two phases. In first phase, the prevailing SW management practices in the MCJ have been evaluated *vis-a-vis* the standard SWM methods, and suggestions have been put forward keeping in mind the ground realities and system limitations. In the second phase, the health impact assessment has been performed in the affected areas using the survey technique.

### Recommendations to modernize the waste management practices prevailing in MCJ

It has been observed during the course of the study that the prevailing SWM practices and boundary conditions are different for urban and rural regions of MCJ and hence the modernization strategies for the two are also different accordingly.

**Recommendations for urban areas:** The problem of preliminary storage of SW is increasing as the population is growing rapidly. The old methods of SW management are not correct. SW should be disposed off after separation into various parts.

- Biodegradable waste and
- Recyclable waste (i.e., plastic, metal, glass, leather) etc.

People should be made aware of collecting the waste in different bins, i.e. organic and recyclable.

1. Waste should be divided into two parts: Biodegradable and nonbiodegradable. Waste from the hospital should be collected daily and disposed off to the incineration plant.
2. Chemical, pesticides, batteries and other domestic hazardous/toxic waste material should be collected in different bins.
3. Each area should be given to a particular sweeper for the door to door collection of waste. In densely populated areas, about 400 m of road length is given to a particular sweeper. In less-populated areas, about 600 m of road length should be given to a sweeper. Sweepers should collect the SW at one fixed time.

Collection of recyclable/nonbiodegradable waste by NGOs should be motivated to collect the recyclable waste, such as polythene, plastic, glass, metals, so that these can be recycled. Particular residential areas should be given on lease depending on the work of the NGOs.

### Public awareness

SW generally comes from the residential and commercial areas, for example houses, vegetable markets, hotels, marriage palaces, hospitals, institutions, etc. The public should be made aware by arranging awareness camps that the waste should not be spread on streets, roads, nalis, etc. People should be made aware of the fact that if the waste is properly disposed off from the house then the environmental will not get polluted. Many severe diseases can spread by improper disposal of SW. There should be environmental engineers and public health engineers for the SW management in addition to Health Officers related to community medicines. Qualified engineers will work to overcome the drawbacks of this system. There should be trained collectors who know all the details that required for collecting SW from door to door and from streets, roads, etc.[21]

There should be literacy classes in which they learn how the SW can lead to various problems and diseases and how these problems are reduced. There should be health check up camps of community waste collectors. The number of engineers and other staff members should be adequate according to the population of the area, provision of bins, containers, rickshaw and trolleys and trucks. Their number should be sufficient and the government. should take care that the number of these equipments and material of the containers should be okay. Proper finances and system to system coordination is an important factor.

### Health education

Health education is major part of the control programme of these diseases. Environmental awareness, i.e. impact of various environmental factors on human beings, is yet another important factor that must be addressed. The health worker and doctors should tell people about the common diseases against which care should be taken. Preventive measures should be told to people and they should be told to covers all the eatables. Boiled water should be used for drinking purposes. Hand should be washed before eating anything. Ammunition schedule should be followed by the people. People should be made aware of population control. Basic contraception methods should be told to the people. Doctors and environmentalists should arrange awareness camps.
